# Evaluation of Characteristics and Building Applications of Multi-Recycled Concrete Aggregates from Precast Concrete Rejects

**DOI:** 10.3390/ma15165714

**Published:** 2022-08-19

**Authors:** Ángel Salesa, Luis M. Esteban, Pedro Luis Lopez-Julian, José Ángel Pérez-Benedicto, Alejandro Acero-Oliete, Alfredo Pons-Ruiz

**Affiliations:** 1 Materials and Structures Department, Engineering School of La Almunia (EUPLA), Universidad de Zaragoza, La Almunia de Doña Godina, 50100 Zaragoza, Spain; 2 Applied Mathematics Department, Engineering School of La Almunia (EUPLA), Universidad de Zaragoza, La Almunia de Doña Godina, 50100 Zaragoza, Spain; 3 Group of Hydraulics and Environmental Engineering (GIHA), Engineering School of La Almunia (EUPLA), Universidad de Zaragoza, La Almunia de Doña Godina, 50100 Zaragoza, Spain

**Keywords:** construction and demolition waste, multi-recycled concrete aggregates, green road construction, recycled concrete, physico-mechanical properties

## Abstract

The construction industry must meet current environmental requirements, mostly those pertaining to the reduction in construction and demolition waste and the consumption of raw materials. The use of recycled concrete aggregates can be part of the solution, but one question that arises is how many times recyclables can be recycled. This unknown involves other related queries regarding the properties and possible uses of repeated recycled concrete aggregates. This research is derived from the precast concrete industry, where multi-recycling is a pressing need. From good-quality parent concretes, three cycles of recycled concrete aggregates were produced and analysed. The final results are promising due to the good quality of the recycled and multi-recycled concrete aggregates obtained. Not only can they be used in low-level applications (backfilling) as usual, but they can also be used for more demanding purposes, such as graded aggregates, cement-treated road bases and concrete pavements. Their use in structural concrete is feasible, but it will be dependent on the water absorption level and the amount of recycled aggregate substitution. This research proves the viability of multi-recycled concrete aggregates with all of the associated environmental benefits.

## 1. Introduction

The construction sector is one of the largest polluters on earth, as it consumes large quantities of raw materials and energy [1]. Among these raw materials, aggregates (fine and coarse) are the most essential components of construction. They are used in a multitude of applications (road base, railway ballast, filling, etc.) and products (concrete, bricks, asphalt, etc.). According to the European Aggregates Association [2], in 2019 in the European Union, the total production of aggregates was estimated to be 2826 million tons, and just 273 (9.7%) came from recycling and reusing.

One of the consequences of this exacerbated consumption is the huge generation of construction and demolition waste (once a structure has reached the end of its lifespan, there is a change in its function or there are structural failures). Due to the size of the construction sector and its raw material dependency, construction and demolition waste (CDW) is the principal waste stream by volume in the European Union (EU), about one-third of all the waste produced [3].

The European legislation [4] requires Member States to achieve a 70% CDW recovery rate by 2020. According to the European Environment Agency, EU countries are on track to fulfil the 70% recovery target set for the year 2020. However, not all that glitters is gold since EU states mostly achieved this figure by using CDW for low-grade recovery applications and backfilling. [5]. Moreover, despite the fact that dumping is the most inadequate action in CDW treatment and should be avoided, a significant percentage of CDW is landfilled [6].

Every year, billions of tons of CDW is dumped into landfill sites worldwide, causing corresponding social, economic and environmental problems [7,8]. China, in 2018, generated more than 2.36 billion tons of CDW [9], and its recycling rate is only about 5% [10]. In the EU, this figure exceeds 850 million tons [11], as some of its countries still have high landfilling rates: 47% in Slovakia, 43% in Cyprus, 30 % in France, 24% in Sweden, 24% in Croatia and 21% in Spain [5]. In the USA, in 2018, 600 million tons of CDW was generated, 455 million tons was directed to the next use, and 145 million tons (24.1%) was sent to landfills [12]. Other countries also have significant landfilling rates: in India, landfilling and open dumping along roadsides and in water bodies are the prevailing management practices for its 150 million tons of CDW [13]; in Brazil, around 92% of the collected CDW is not recycled or stored for future use (inert landfill) [14]; and in Australia, 27% of the generated CDW is landfilled [15].

Currently, our world lives on war images. These unfortunate events especially affect people but also ruin infrastructures and buildings, generating vast amounts of CDW. From World War II [16] until the most recent armed conflicts [17,18,19,20,21], the correct management and reutilisation of rubble and debris have been priorities in post-conflict reconstruction policies.

In light of the previous figures and circumstances, and bearing in mind that most CDW is economically and environmentally reusable and recyclable [1], the loss for the circular economy is tremendously high. Despite the magnitude of the problem, it can be stated that CDW materials are not effectively managed worldwide [7], and, consequently, it is necessary to move towards truly circular waste management [5] that provides ecological and sustainable benefits [22,23].

Some of the reasons explaining this inappropriate management of CDW may be due to uncompetitive pricing, lack of trust in the quality of secondary materials, lack of information on the composition of materials used in existing buildings and the long delay between the passing of new CDW management laws and their effective implementation [5]. Contrary to what it might seem, there is no correlation between landfill taxation and the percentage of CDW landfilled or recovered; according to Villoria et Osmani [11] and Reis et al. [24], the key CDW recovery challenges are: ineffective CDW regulations, lack of standardised tests, poor data quality and harmonisation, poor reverse logistics and low market readiness for secondary materials.

As has been seen, the disposal of CDW is still a current problem in many countries. CDW can be recycled and reused as garden pavement, gabions, road and train base layers, ground improvement applications, recycled concrete, recycled bricks, etc. Nevertheless, their consumption is still limited compared to the generated quantity of CDW [25].

In the last decades, it has been proved that CDW, with adequate treatment (separation, crushing, classification and cleaning), can be transformed into recycled aggregates (RAs) to replace natural aggregates (NAs) in new structures. This action can provide benefits for the environment and economy [25]. According to Hossain et al. [26], recycled coarse aggregates produced from CDW, compared to natural coarse aggregates, reduce 65% of greenhouse gas emissions with a saving of 58% non-renewable energy consumption. Similar environmental benefits were observed for recycled fine aggregates. Regarding the economic aspect, Silva et al. [27] stated that in a road pavement renovation, RA offered an immediate cost saving of about 0.50 GBP/m^2^ compared to conventional NA; Ohemeng and Ekolu [28] pointed out that the production of recycled concrete aggregates is less expensive than that of natural aggregates (the long-term cost was 40% less).

CDW can include ceramic particles, mortar, concrete, natural aggregates, asphaltic material, gypsum, ceramics, wood, metal particles, glass, paper and plastic. Therefore, recycled aggregates from CDW can be highly heterogeneous. Obviously, this heterogeneity and the characteristics of recycled aggregates depend on the nature of CDW constituents [29]. Thus, the application of a selective demolition process is of great importance for the ability to adequately characterise RAs, thereby increasing the quality of recycled aggregates [30].

Two main categories of recycled aggregate (RA) can be established: recycled concrete aggregate (RCA), coming mainly from concrete waste, and mixed recycled aggregate (MRA) from diverse waste source materials [22].

One of the key aims of CDW management is the use of RA in highly demanding applications, such as the manufacture of recycled concrete (RC). For this purpose, RCAs are mainly used, excluding the use of MRAs.

The viability of using recycled aggregates in concrete production has been repeatedly validated with satisfactory results in mechanical performance, especially with coarse recycled concrete aggregates [31,32,33,34,35] but also with fine concrete recycled aggregates [1,36,37].

As RA influences the physical and mechanical properties of RC, for concrete production and other high-grade applications, RCA from a high-quality parent concrete is desirable. The precast concrete industry can serve as one of the most suitable recycled aggregate sources due to its quality control, traceability and common use of top-class concrete components [38,39,40,41].

Prefabrication is the industrialised version of construction, and it has many advantages in comparison to traditional construction: higher reliability and quality, higher dimensional precision, optimisation of sections, better work safety and a longer service life [42,43,44,45]. For these reasons, in recent decades and in many countries, the prefabrication construction industry has become increasingly popular, consolidating the use of precast concrete elements [46,47].

In the near future, some of the issues that we will have to address regarding CDW management are as follows: What happens to new concrete structures made with recycled aggregates once these structures have reached the end of their service life? Is it possible to demolish them and produce recycled concrete aggregates again? What will be the characteristics of these multi-recycled concrete aggregates? Could they be used for producing new recycled concrete? Or could they be used in other construction applications?

In the precast concrete industry, the above questions are not future but current issues. The precast industry generates a large amount of concrete waste every year due to discrepancies in quality controls [48]. In Spain, in 2018, 4.6 million tons of precast concrete pieces was produced; assuming a manufacturing rejection ratio of 3–6% [49,50], between 138,000 and 276,000 tons of concrete waste was generated in the Spanish precast concrete industry [38]. These values provide a relevant sign of the problem size, since the rejected pieces, instead of being crushed and later used as recycled aggregates, are commonly deposited in landfills.

The construction industry does not rapidly incorporate technical advances. It needs a successful history of use together with the existence of guidelines or codes that support the use of new materials or techniques [27,51]. Hence, although multi-recycled concrete aggregate has been partially studied in the last few years [31,48,52,53,54,55,56,57,58,59,60], the building industry and, more specifically, the precast industry, need to increase the amount of practical knowledge research to promote the use of multi-recycled concrete aggregate.

The present research on multi-recycled concrete aggregate was developed with the purpose of increasing the technical knowledge of this particular type of aggregate and, more particularly, identifying more demanding construction applications beyond the typical backfilling.

Based on the obtained results, repeated recycled concrete aggregate can become a significant source of aggregates in construction roads and concrete manufacturing, avoiding the need for mining and debris landfilling, with all of the consequent potential environmental and economic benefits.

## 2. Literature Review

### 2.1. Multi-Recycled Concrete Aggregates

The main difference between natural aggregates (NAs) and recycled aggregates (RAs) is that the second type is made up of both debris of natural aggregates and cement hydration products (cement mortar) [32,38,61].

The attached mortar lends RA different characteristics from NA; the main modifications are: a higher porosity, higher water absorption, a lower density and a lower elastic modulus [39,40,52,56]. These changes obviously affect the properties of the pieces or products fabricated with RA instead of NA [57].

The multi-recycling process amplifies changes in the characteristics of RA and, consequently, in the properties of products made. It is accepted that the properties of RA usually worsen as the number of recycling cycles increases because the amount of adhered mortar also increases [31,48,52].

RAs have a lower density than NAs, and it decreases with each recycling loop since, with each recycled sequence, the amount of adhered mortar increases [55,57,59,60,62], and mortar tends to have a lower density than NA. The amount of adhered mortar in RA increases until the presence of the original natural aggregate is negligible, which is the case from the fourth cycle on [58].

The larger amount of mortar, the higher the water absorption. Therefore, RAs have higher water absorption than NAs. Moreover, the porosity of RA is higher, and it helps fluid penetration. The greater the number of recycling cycles, the higher the water absorption of RA [48,55,58]. With the first recycling cycle, the density decrease and porosity and absorption increase are significantly greater than in consecutive recycling cycles [58].

The crushing process also has a great influence on RAs, mainly on their angularity. It has been found that RAs tend to present a more angular shape than NAs [63]. Crush resistance and rupture resistance decrease with each recycling repetition [60]. Regarding fragmentation resistance, the Los Angeles test is the most common method for measuring toughness, degradation and abrasion resistance. Most studies stated that resistance to crushing and abrasion of RA is lower than that of NA; i.e., RAs have a higher Los Angeles index [52]. As happens with other properties, the Los Angeles index tends to increase with each recycling stage [31].

The characteristics of parent concrete have a direct influence on the quality of the RA obtained [52]. Regarding the number of recycling cycles and aggregate characteristics, three cycles were not enough to achieve the stabilisation of aggregate properties (apart from Los Angeles Abrasion) [57].

### 2.2. Multi-Recycled Concrete Aggregates for Concrete Production

One of the most ambitious objectives of recycling CDW is to use recycled aggregates to produce recycled concrete (RC) without significantly affecting the properties and durability of concrete. For this purpose, recycled concrete aggregates (RCAs) are mainly used, and mixed recycled aggregates (MRAs) are excluded.

Regarding the properties of RC that has been repeatedly recycled, and according to Grabiec et al. [64], there is little discussion about multi-recycled concrete (MRC) properties since there is not enough data to discuss them in spite of the research efforts in recent years.

**Figure 1:**
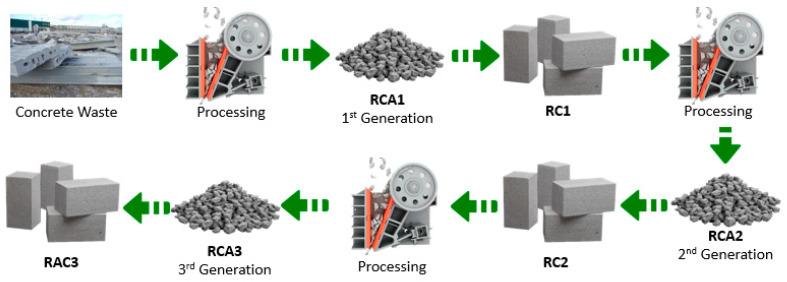
Scheme of repeated use of RCA in repeated RC manufacturing.

It should be taken into account that RC is influenced by: first, the properties of RCA, which depend on the typology of the “parent concrete”; second, the percentage of replacement of NA by RA [65]. The main characteristics of the repeated use of RCA in the manufacturing of MRC (Figure 1) are the following:

- Density and water absorption: The density of the first, second and successive RC decreases, while the water absorption increases [31,48,56,57,59,60,66]. For Huda and Alam [55], the decrease in density was 10% and 14% for RC1 and RC2, whereas water absorption increased by 1.9% and 4.2%. For Salesa et al. [40], the dry density decreased by 0.3%, 1.19% and 3.6%, and the water absorption increased by 11.6%, 17% and 20.3% for RC1, RC2 and RC3, respectively.
- Workability of fresh concrete: The workability of MRC decreases with each recycling sequence, which is due to the higher water absorption of RCA in comparison to NA. For Salesa et al. [39], the workability dropped by 20.3% (RC1) and 27.5% (RC2). A similar trend was stated by other authors [31,55,56,60,66].
- Compressive strength: The compressive strength of MRC tends to decrease as the number of recycling repetitions increases [55]. For Zhu et al. [60], the reduction for a replacement ratio of 70% of coarse aggregate was 6.7% (RC1), 16.6% (RC2) and 25.18% (RC3). For Abreu et al. [31], with a substitution ratio of 100% of coarse aggregate, the decrease was 3.2% (RC1), 4.7% (RC2) and 13.1% (RC3).
- Conversely, when the quality of RCA is high, an improvement in the compressive strength can be observed [41,67]; e.g., Salesa et al. [40] reported that for a 100% coarse aggregate substitution ratio, the increase was 7.7% (RC1), 10.9% (RC2) and 13.8% (RC3).
- Tensile strength: The tensile strength also tends to decrease with each recycling repetition [54,56,60]. For Abreu et al. [31], with a substitution ratio of 100% of coarse aggregate, the decrease was 9.1% (RC1), 11.4% (RC2) and 15.1% (RC3).
- Elastic modulus: Since cement mortar usually has a lower elastic modulus than natural aggregate, the elastic modulus of MRC tends to decrease with each recycling series [31,54,55]. Salesa et al. [40] reported a decrease of 4.3% (RC1), 7.5% (RC2) and 13.8% (RC3). This entails greater mid-span deflections in beams made with RCA instead of NA [68].
- Attached mortar: According to Zhu et al. [60], Thomas et al. [58], Abet et al. [66] and Silva et al. [57], the amount of attached mortar in MRC increases as the number of recycling repetitions increases.
- Durability: In an extensive experimental campaign, Silva et al. [57] stated that with the increase in recycling cycles, recycled coarse aggregates show a quality decrease in their properties, resulting in worse durability and shrinkage performance of the resulting concrete. Regarding certain durability factors, the open and closed porosity and sorptivity increase with each recycling cycle, and chloride permeability also rises [48,59]. Despite the durability decline, MRC can meet design life durability requirements [69,70].

### 2.3. Multi-Recycled Concrete Aggregates for Other Applications

Although RCA can be technically used for RC manufacturing, it is mainly used in secondary applications for road subbases and bases, railway capping layers and backfilling [64,71,72].

The good performance of RCA as granular material applied to road base layers has been proved in several studies [73,74,75,76,77]. Its use is even feasible in concrete pavement construction [33] as long as RCA is of good physical–mechanical quality [78]. Moreover, RCA can effectively replace NA in cement-stabilised pavement bases [79]. There is already satisfactory evidence for the use of multi-recycled concrete aggregates in road base construction [80].

In actual fact, some public administrations have developed standards, codes or recommendations for encouraging the use of recycled aggregates in road construction [81,82]

Likewise, RCA has found a niche application in geotechnical applications. RCA can be an excellent backfilling material (gravelly soil, embankments, stabilised earth walls and pipeline trenches) [83,84,85,86,87,88]. In addition, RCA is suitable for cement replacement for the stabilisation of clay soils, improving compressive strength and reducing deformability and settlement [89,90]. As a granular material, it also has a worthy performance in constructed wetlands and drainage layers [91,92,93].

There are also more complex applications for CDW. One of the most interesting ones is the use of RCA as an alternative source of aluminosilicate precursor in the manufacturing of geopolymeric materials [94,95].

## 3. Regulatory Framework for Recycled Aggregates

The Spanish General Technical Specifications for Roads and Bridge Works (PG-3) [96] only recommend the use of recycled aggregates from CDW in road subbase layers (graded aggregates), cement-bound gravel, cement–soil and lean concrete pavement.

The use of artificial graded aggregate in road construction should follow Article 510 of the Spanish General Technical Specifications for Roads and Bridge Works (PG-3), whose requirements are shown in Table 1. In addition, in Table 1, the requirements for the use of recycled aggregates in cement-bound gravel and cement–soil (Article 513 PG-3) and lean concrete pavement are shown (Articles 550 and 551 PG-3).

It is certainly true that a large number of countries have published regulations in order to increase the amount of RA from CDW in building and road construction. Commonly, these regulations are adapted to the peculiarities of each area, resulting in RA regulations with significant differences [97].

In order to simplify the use of RA for road construction, in 2019, De Brito et al. presented a proposal of an international classification of RA [81]. The classification (Table 2) shows six types of RAs: two for RCA, three for MRA and one for recycled asphalt aggregate (RAA).

Regarding recycled concrete, the Spanish Structural Concrete Code [98] (EHE-08) allows the use of recycled concrete aggregates for the manufacturing of new concrete, but their usage is limited to coarse aggregate substitution of up to 20%. In Table 3, the EHE-08 aggregate requirements for concrete manufacturing for both natural aggregates and recycled aggregates are shown.

In 2020, Bravo et al. [1] developed a technical specification for the use of fine recycled aggregates from construction and demolition waste in concrete production. The minimum requirements for the use of fine particles are shown in Table 4.

## 4. Materials and Methods

This research is focused on the physical and mechanical properties of multi-recycled concrete aggregates. Having repeated RCA is necessary for manufacturing several cycles of recycled concrete. Therefore, in the first subsection, Materials, a description of the repeated concrete manufacturing process and repeated recycled aggregate generation is given. In the second subsection, Performed Tests, the analysis methods carried out in this research are listed.

### 4.1. Materials

**Figure 2:**
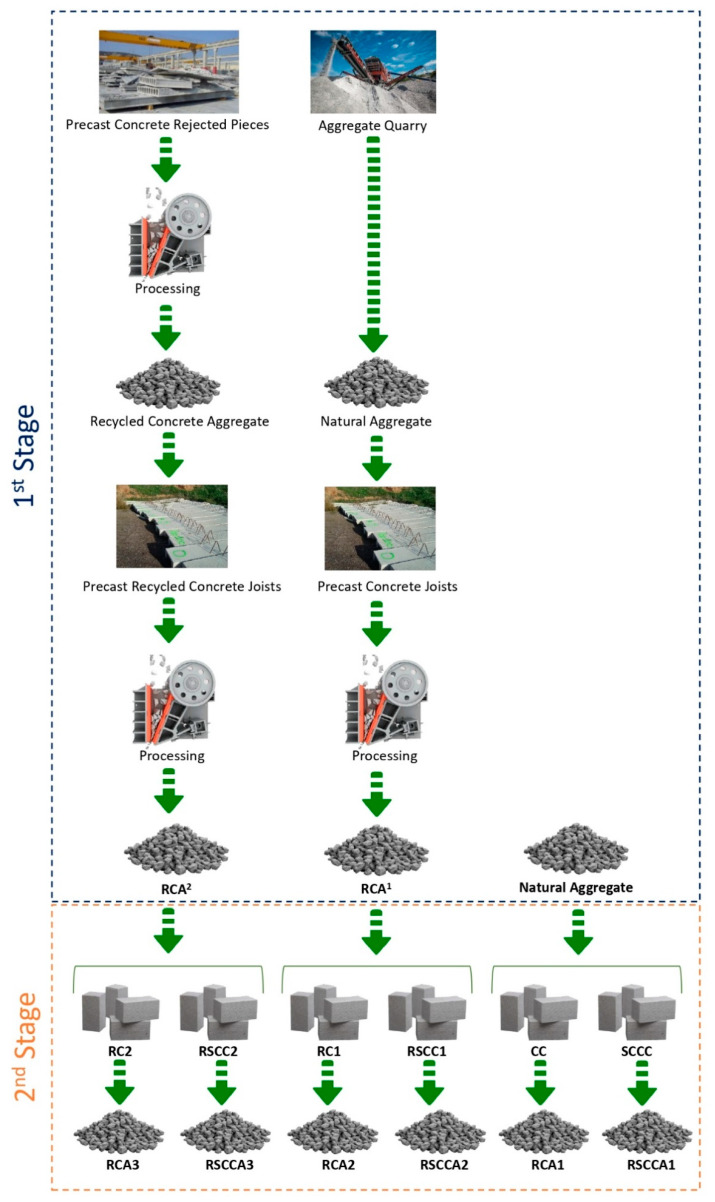
First- and second-stage recycled concretes and aggregates.

The multi-recycled concrete aggregate production in this research is divided into two stages. A graphical summary of both stages is shown in Figure 2.

The first stage of this research started in a precast concrete factory. From rejected concrete precast pieces, excluded for not passing quality controls, RCA was produced. With that RCA, several precast recycled concrete joists with 100% substitution of coarse aggregate were manufactured. Moreover, some precast concrete joists using just NA were also produced. Thereafter, the precast concrete joists were crushed and sieved, and two types of recycled concrete aggregates were generated:

- RCA^1^: the first generation of recycled concrete aggregate from precast concrete joists made with natural aggregates (NAs).
- RCA^2^: the second generation of recycled concrete aggregate from precast recycled concrete joists made with recycled coarse concrete aggregate (RCA) (100% replacement).

The concrete dosage for the production of first-stage precast concrete joists is shown in Table 5.

In the second stage, normal and multi-recycled concretes were manufactured with the coarse fractions from the first stage (RCA^1^, RCA^2^ and NA). Two concrete types were produced: ordinary concrete and self-compacting concrete. In recycled concretes, the natural coarse fraction was 100% substituted by RA. Ordinary concrete dosage was the usual dose used in the concrete industry to produce 25 MPa reinforced concrete, and for self-compacting concrete, a common dosage for producing prestressed self-compacting concrete of 45 MPa was used (Table 6).

Once the new concretes were produced, they were crushed and sieved, obtaining three different fractions (gravel, grit and sand) for analysis. The following types of recycled concrete aggregates and concretes were used and generated, respectively, in this second stage:

- NA: Natural aggregate. Crushed limestone from CEMEX Spain—Quarry of Mezalocha.
- CC: Control concrete made only with NA.
- SCCC: Self-compacting control concrete made only with NA.
- RCA1: First generation of recycled concrete aggregate from CC.
- RSCCA1: First generation of recycled concrete aggregate from SCC.
- RC1: First generation of recycled concrete made with 100% coarse fraction replacement of natural aggregates by RCA^1^.
- RSCC1: First generation of recycled self-compacting concrete made with 100% coarse fraction replacement of natural aggregates by RCA^1^.
- RCA2: Second generation of recycled concrete aggregate from RC1.
- RSCCA2: Second generation of recycled concrete aggregate from RSCC1.
- RC2: Second generation of recycled concrete made with 100% coarse fraction replacement of natural aggregates by RCA^2^.
- RSCC2: Second generation of recycled self-compacting concrete made with 100% coarse fraction replacement of natural aggregates by RCA^2^.
- RCA3: Third generation of recycled concrete aggregate from RC2.
- RSCCA3: Third generation of recycled concrete aggregate from RSCC2.

### 4.2. Performed Tests

For the comparison between the different generations of repeated RCA, several tests were performed. The tests were carried out according to standard procedures. In Table 7, a list of the performed analyses for each size fraction is shown, plus the corresponding standard designations according to which the tests were conducted.

Due to the importance of particle size distribution, particularly in recycled aggregates, particle density and water absorption, these properties were determined for all the aggregates sizes.

Flaky particles in construction are not desirable since if there are loads acting along the thin axis of the flaky flat particles, they can be easily broken down. In order to evaluate whether repeated recycling affects this value, the flakiness index was evaluated for all coarse sizes (gravel and grit).

For fine aggregate sizes, sand equivalent tests were carried out to show the relative proportions of fine dust or clay-like materials in fine aggregates.

Aimed at assessing the abrasion and toughness resistance of aggregates, the Los Angeles test was carried out on coarse fractions (gravel and grit). To evaluate the quality of aggregates that will be subjected to the action of atmospheric agents, specifically the resistance to natural freeze–thaw action, a magnesium sulphate test was performed on gravel-size aggregates.

Regarding the chemical properties of aggregates, for fine-size particles, the total amounts of sulphur (S%), acid-soluble sulphates (SO3s%) and water-soluble chlorides (Cl%) were also evaluated.

## 5. Results and Discussion

The obtained results of the performed tests are presented in Table 8. In the following subsections, these results are analysed.

### 5.1. Size Distribution

**Figure 3:**
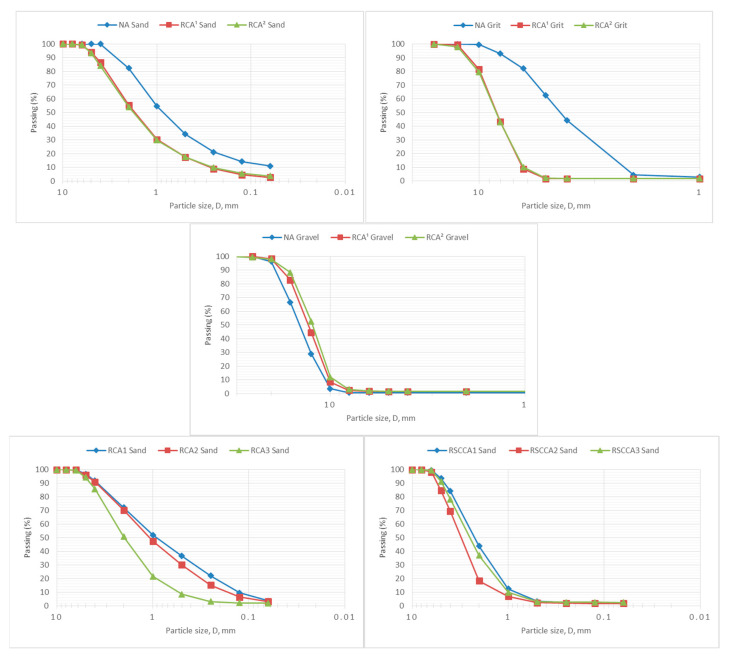

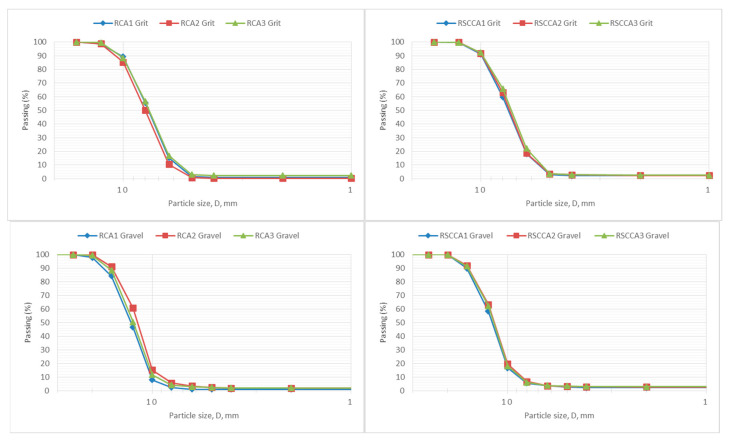
Particle size distribution of different analysed natural and recycled aggregates sizes.

In Figure 3, the different grading curves of both natural aggregates and recycled and repeated recycled aggregates analysed in the study are shown. Particle size distribution is a critical property of aggregates because it dictates particle packing.

Most of the analysed coarse aggregates (grit and gravel) have a d/D ratio higher than 1.4; therefore, it can be stated that the size distribution is well-graded. Similar results were obtained in previous research [60]. There are no significant differences between the coarse sizes of the different recycling cycles, neither in recycled self-compacting concrete aggregates nor in recycled common concrete aggregates.

Regarding the fine aggregates (sand), NA sand shows a typical S-shape distribution [36]. RCA^1^ and RCA^2^ sands also display an S-shape distribution but with larger particle sizes. Regarding sands of the second stage, their size distribution tends to be a bit more evenly graded than NA sand, but still well-graded. It can also be observed that with each repetition of the recycling process, a bigger particle size distribution was obtained, which is contrary to some previous research [99,100] but in accordance with other studies [55,60]. This can be explained by the high quality of parent concretes (dosage and careful manufacturing), which causes larger sizes due to their hardness in the crushing process.

Regarding the number of particles < 0.063, recycled coarse aggregates (grit and gravel) have, in general, a number of fine particles higher than 1.5% (limit value of EHE-08); only some of the first recycled cycle aggregates have values below this limit. To meet the fine quantity specification, the coarse recycled aggregates should be washed. With each recycling cycle, the number of fine particles attached to coarse aggregates increases. However, recycled sands have values between 1.6 and 3.5, lower than 6%, which is the limit value of EHE-08.

### 5.2. Sand Equivalent Test

The sand equivalent test shows that with each recycling cycle, the cleanliness of fine aggregates tends to be higher: the test results increase with each cycle. Between the third and second cycles, the difference is greater than between the first and second recycling cycles.

In all of the recycled sands, the sand equivalent index is higher than 75, which is the minimum value for the worst concrete exposure classes, according to EHE-08. Moreover, the obtained results allow the use of recycled sands as graded aggregate, cement-bound gravel and cement–soil, according to the limit of > 40 by PG-3. Considering the above, it can be stated that recycled and multi-recycled sands show good quality in relation to the presence of silt and clay.

### 5.3. Flakiness Index

The presence of flaky and elongated aggregate particles is considered undesirable for aggregate construction applications due to their weakness and the possibility of breaking down under loading. All of the recycled and multi-recycled aggregates showed small values of the flakiness index, which is good. A trend in the flakiness index with the number of recycling cycles was not found. The same was observed in other studies [31]. Differences between self-compacting concrete and common concrete aggregate origin were found.

In all of the analysed recycled coarse aggregates, the flakiness index is lower than 8, which is far below the limit of 35 for concrete production (EHE-08) and of the 30–35 requirement for graded aggregate, cement-bound gravel and cement–soil (PG-3).

### 5.4. Density and Water Absorption

The density of a recycled concrete aggregate directly depends on the density of the parent concrete. All of the recycled aggregates analysed have a density higher than 2000 kg/m^3^.

It can be seen that within the natural aggregate, the highest density corresponds to the gravel size, and it decreases when particle size decreases. This supports the statement that the size of the particles influences the density. Smaller fractions have lower density. However, in the recycled aggregates, a different trend is found, as the highest density tends to occur in the grit-size aggregates. A similar outcome was found by Thomas et al. [58].

The density of recycled aggregates is directly related to the amount of adhered mortar that they have since their density is lower than that of natural aggregates. For aggregates from ordinary concrete, the decrements were 11, 12 and 15 % for gravel and 7.5, 9 and 11.4% for grit for the first, second and third recycling cycles, respectively. For aggregates from self-compacting concrete, the decrements were 14, 14 and 16 % for gravel and 11, 10 and 12.5% for grit for the first, second and third recycling cycles, respectively. In light of the above, density decreases with each recycling cycle.

Previous multi-recycled concrete aggregate research [31,48,55,57,58,60] also stated that density decreases with each recycling cycle. The average density decrease was 8.9%, 13.7% and 17.5% for the first, second and third recycling cycles, respectively. Literature figures are in line with the ones obtained in this research. According to the above figures and results, it can be stated that density decreases with each recycling cycle, but the differences between cycles tend to be lower with each loop, with the first one being the most notable.

Density and water absorption are closely bound since the amount of adhered mortar, which reduces particle density, increases water absorption.

Recycled aggregate water absorption is much higher than natural aggregate absorption. Apart from one, all of the recycled coarse aggregates have a water absorption higher than 5%, which is the absorption limit for concrete manufacturing according to EHE-08. However, when the natural aggregate replacement ratio is lower than 20%, the water absorption limit is 7%. In this case, all coarse recycled aggregates meet requirements, with the exception of the RSCCA3 gravel.

In natural aggregates, water absorption increases when aggregate size decreases, but in recycled aggregates, due to the amount of adhered mortar in larger particles, some grit particles have lower absorption than gravel particles. On average, for ordinary concrete aggregates, the absorption was 4.7, 6.2 and 6.5% for RCA1, RCA2 and RC3, respectively. For self-compacting concrete, absorption was 6.4, 6.5 and 7.13% for RSCCA1, RSCCA2 and RSCCA3, respectively. Literature results [31,48,55,57,58,60] also show a similar tendency; the average absorption results were 5.5, 7.95 and 9.6% for the first, second and third recycling cycles. According to the above, it can be stated that water absorption increases with the repetition of recycling.

### 5.5. Resistance to Fragmentation

To assess the abrasion resistance of aggregates, the Los Angeles test was performed. The Los Angeles index gives an indication of the quality and competence of any type of aggregate, where a higher index means worse abrasion resistance.

In the first stage of the study, the LA index was 24 and 21 for RCA^1^ gravel and grit and 28 and 24 for RCA^2^ gravel and grit. It can be observed that gravel-size particles have a worse LA index than grit size particles; additionally, with each recycling cycle, the abrasion properties tend to become slightly worse.

In the second stage of the study, the LA index for ordinary concrete was 35 and 33, 30 and 27, and 32 and 28 for gravel and grit of RCA1, RCA2 and RCA3, respectively. For the self-compacting concrete, the results were 36 and 33, 32 and 27, and 34 and 29 for RSCCA1, RSCCA2 and RSCCA3 gravel and grit, respectively. In this case, the LA index does not show a clear evolution trend with each recycling cycle since the second recycling cycle results are better than the first and third recycling cycles.

Analysing previous related research [31,57,58], the average results were 38, 41 and 41% for the first, second and third recycling cycles. Our aggregates have a better fragmentation performance than literature figures, and this could be due to the high quality of parent concretes. In addition, in the literature, a clear trend was not found with the increase in the number of recycling loops.

In all of the recycled and repeated recycled cases, the LA index values are far below the EHE-08 limit of 40 for concrete manufacturing. However, for their use in road construction, not all of the aggregates meet the LA requirements of PG-3. The aggregates of the first stage (RCA^1^ and RCA^2^) comply with the limits, but the RCA of the second stage does not in all cases. RCA1 and RSCCA1 can only be used as coarse aggregates in cement-bound gravel and cement–soil layers for road shoulders. RCA2, RCA3, RSCCA2 and RSCCA3 can be used for cement-bound gravel and cement–soil layers as graded aggregate and for concrete pavement for low-traffic-intensity roads.

### 5.6. Thermal and Chemical Properties

Regarding the thermal resistance of recycled aggregates, the magnesium sulphate test on gravel-size aggregates showed results within a range of <2–4%. These values are far below the EHE-08 and PG-3 graded aggregate limit of 18%.

In relation to chemical properties, recycled aggregates show water-soluble chloride values (Cl%) of <0.006, which largely satisfies the limit value of 0.03 for concrete production established by EHE-08.

As for the amount of sulphur content (S%) and acid-soluble sulphates (SO3s%), recycled aggregates had values of ≤0.2% and <0.8%, respectively; in both cases, recycled aggregates meet the requirements for concrete production (EHE-08, with limits of 0.4% for S% and 0.8 for SO3s%). They also meet regulations for the production of graded aggregate, cement-bound gravel and cement–soil (PG-3). No significant differences between either the types of parent concrete (self-compacting and normal concrete) or aggregate sizes were found.

### 5.7. Summary

Some physical, mechanical and chemical properties of recycled and multi-recycled concrete aggregates are analysed and discussed above. Likewise, they are compared with Spanish road construction regulations, Spanish concrete guidelines’ requirements and other international guidelines’ specifications.

Most of the properties meet the guidelines’ requirements. The most restrictive factor is water absorption, limited to 5–7% for concrete manufacturing, which is exceeded in some aggregate types. To solve this problem, the percentage of recycled aggregate substitution should be limited to 20% of the coarse fraction, or some pre-processes should be carried out, such as pre-treatments or pre-saturation.

The obtained recycled and multi-recycled concrete aggregates’ resistance to fragmentation was suitable for their use as aggregates for structural concrete manufacturing. In almost all cases, the recycled and multi-recycled aggregates also showed good results for their use as graded aggregate, concrete pavement and cement-treated road bases.

The coarse recycled and multi-recycled aggregates of this study were shown to have satisfactory physical, mechanical and chemical properties for their use in concrete manufacturing and road construction according to Spanish regulations (EHE-08 and PG-3). Regarding fine recycled and multi-recycled aggregates, in all cases, the quality was good enough, and all of the obtained aggregates could be classified as “Fine RA Type I” according to Bravo et al.’s [1] specification proposal. This category would allow their use in concrete manufacturing. The positive sand equivalent test index results will also enable the use of recycled and multi-recycled fine fractions as part of graded aggregate, cement-treated road bases and concrete pavements.

## 6. Conclusions

On the basis of the results obtained, the following conclusions can finally be drawn:

- Recycled and multi-recycled concrete aggregates with up to three recycling loops can be used as graded aggregates, in concrete pavement and in cement-treated bases for road construction. They meet the established requirements of codes and guidelines (PG-3, Bravo et al.’s classification proposal [1] and Brito et al.’s regulation proposal [81]).
- Recycled and multi-recycled concrete aggregates with up to three recycling loops could be used for structural concrete manufacturing, provided that the water absorption of recycled aggregates is taken into account and amended with pre-treatments or pre-saturations when needed.
- It has been proved that good-quality parent concretes provide high-grade recycled and multi-recycled aggregates that can be used not only in low-level applications but also in more demanding tasks.
- Concrete aggregate multi-recycling is feasible, and it will contribute to a reduction in natural raw material consumption, decreasing the ecological footprint.

## Figures and Tables

**Table 1:** PG-3 technical requirements for graded aggregate, cement-bound gravel and lean concrete pavement [96].

Performed Test	Graded Aggregate (Article 510 PG-3)		Cement-Bound Gravel and Cement–Soil (Article 513 PG-3)		Concrete Pavement and Vibrated Lean Concrete Pavement (Articles 550 and 551 PG-3)	
Title	Coarse Aggregate	Fine Aggregate	Coarse Aggregate	Fine Aggregate	Coarse Aggregate	Fine Aggregate
Determination of particle size distribution	X	X	X	X	X	X
Flakiness index	<35		<30–40		<35	
Sand equivalent test index		>40–30		>40–35		>70–75
Los Angeles test index (LA)	<30–35		<30–40		<35	
Particle density						
Water absorption						
Magnesium sulphate test	<18%					
Water-soluble chloride salts (Cl%)		X				
Total sulphur content (S%)	<0.5%–1%	<0.5%–1%	<1%	<1%		
Acid-soluble sulphates (SO3s%)	<0.7%	<0.7%	<0.8%	<0.8%		

**Table 2:** Classification of RAs for international application in road sections [81].

Type of RA	Composition				Min. Density (SSD)	Water Abs. (%)	Los Angeles (%)	Water-Soluble Sulphate (%)	Proposed Applications
	Rc + Ru (%)	Rb (%)	Ra (%)	Others (%)					
RCA-I	>90	<10	<5	<1	2200	<6	<35	<0.7	Concrete pavement, cement-treated or unbound subbases
RCA-II	>85	<15	<10	<3	2100	<8	<37	<0.8	Cement-treated or unbound subbases
MRA-I	>70	<30	<10	<5	1900	<8	<40	<0.8	Unbound granular subbases or capping of esplanades
MRA-II	>60	<40	<20	<8	1800	<12	<45	<1.0	Capping of esplanades or subgrades
MRA-III	>40	<60	<30	<15	1650	<15	<50	<1.2	Subgrades and embankment
RAA	<50	<10	>50	<3	2000	<8	<40	<0.8	Unbound granular subbases or capping of esplanades

Rc: concrete and natural aggregates with adhered mortar; Ru: particles of natural materials such as rocks, gravel, etc.; Rb: particles of ceramic, bricks, tiles, calcium silicate masonry units, etc.; Ra: bituminous mixture particles; Others: wood, plastic, plaster, aluminium, etc.

**Table 3:** EHE-08 technical requirements for aggregates for concrete manufacture [98].

Performed Test	General Requirements EHE-08		Additional Requirements for RCA Annex 15 | EHE-08
Title	Coarse Aggregate	Fine Aggregate	Coarse Aggregate
Determination of particle size distribution—sieving method	X	X	
Amount of particles < 0.063 mm (%)	≤1.5%	≤6–16%	
Flakiness index	<35		
Sand equivalent test index		>70–75	
Los Angeles test index	≤40		≤40
Particle density			
Water absorption	≤5%	≤5%	≤7%
Magnesium sulphate test	≤18%		≤18%
Water-soluble chloride salts (Cl%)	≤0.03–0.05%	≤0.03–0.05%	
Total sulphur content (S%)	≤0.4%	≤0.4	
Acid-soluble sulphates (SO3s%)	≤0.8%	≤0.8%	

**Table 4:** Properties and minimum conformity requirements for the use of fine recycled aggregates [1].

Properties	Conformity Requirements
Particle density (kg/m^3^)	≥2000
Water absorption (%)	≤14
Water-soluble sulphate content (%)	≤0.2
Acid-soluble sulphate content (%)	≤0.8
Total sulphur content (%)	≤1.0

**Table 5:** Mix proportions for first-stage concretes.

Proportions in kg/m^3^					w/c Ratio
Coarse Aggregate	Fine Aggregates		Cement	Admixtures (Litres)	
	Sand	Filler			
890	860	100	420 CX CEM I 52.5R	6.2 P-180	0.41

**Table 6:** Mix proportions for second-stage concretes.

Concrete Type	Proportions in kg/m^3^							w/c Ratio
	Coarse Aggregate		Fine Aggregates		Cement	Admixtures (Litres)		
	Gravel	Grit	Sand	Filler				
Ordinary concrete	750	175	1000	-	275 CX CEM II/A-V 42,5R	2.75 CX Isoplast 006	2.75 CX Isoflex 003	0.6
Self-compacting concrete	-	700	1025	75	390 CX CEM I 52.5R	5.85 CX Isocast 951	-	0.5

**Table 7:** Performed tests.

Performed Tests			Size Distribution		
Title	Index	Standard Code Designation	Gravel	Grit	Sand
Determination of particle size distribution—sieving method	-	UNE–EN 933-1	X	X	X
Amount of particles < 0.063 mm	%	UNE–EN 933-1			
Determination of particle shape—flakiness index	FI%	UNE–EN 933-3	X	X	
Assessment of fines—sand equivalent test	SE4	UNE-EN 933-8			X
Los Angeles test—resistance to fragmentation	LA%	UNE-EN 1097-2	X	X	
Determination of particle density	g/cm^3^	UNE-EN 1097-6	X	X	X
Water absorption	Ab%	UNE-EN 1097-6			
Thermal and weathering properties—magnesium sulphate test	MS%	UNE-EN-1367-2	X		
Water-soluble chloride salts by potentiometry	Cl%	UNE-EN-1744-1 Part 8			X
Total sulphur content by high-temperature combustion	S%	UNE-EN-1744-1 Part 11.2			X
Determination of acid-soluble sulphates	SO3s%	UNE-EN-1744-1 Part 12			X

**Table 8:** Summary of experimental results of performed tests on recycled and multi-recycled concrete aggregates.

	Type	Size	Test Results									
			<0.063%	FI%	SE4	LA%	g/cm^3^	Ab%	MS%	Cl%	S%	SO3s%
**FIRST STAGE**	**RCA^1^**	**Gravel**	1.5 ± 0.4	<4	-	24 ± 1	2.33	5.2 ± 0.4	<2	<0.006	<0.2	<0.5
		**Grit**	1.3 ± 0.3	<4	-	21 ± 1	2.38	4.4 ± 0.4	<2	<0.006	<0.2	<0.5
		**Sand**	2.7 ± 0.5	-	93 ± 5	-	2.18	6.2 ± 0.1	-	<0.006	<0.2	<0.5
	**RCA^2^**	**Gravel**	2.0 ± 0.4	<3	-	28 ± 1	2.18	6.6 ± 0.4	<2	<0.002	0.3 ± 0.1	<0.7
		**Grit**	1.8 ± 0.4	4 ± 2	-	24 ± 1	2.25	6.0 ± 0.4	<2	<0.001	0.2 ± 0.1	<0.6
		**Sand**	3.4 ± 0.5	-	79 ± 4	-	2.23	7.0 ± 0.1	-	<0.002	0.1 ± 0.1	<0.6
**SECOND STAGE**	**NA**	**Gravel**	0.6 ± 0.2	<8	-	32 ± 1	2.60	1.1 ± 0.4	4 ± 1	<0.006	NA	NA
		**Grit**	2.8 ± 0.5	12 ± 2	-	31 ± 1	2.54	2.2 ± 0.1	4 ± 1	<0.006	<0.1	<0.2
		**Sand**	10.8 ± 0.9	-	73 ± 4	-	2.44	2.5 ± 0.1	-	<0.006	<0.1	<0.2
	**RCA1**	**Gravel**	0.8 ± 0.3	<8	-	35 ± 1	2.34	4.6 ± 0.4	NA	NA	NA	NA
		**Grit**	0.6 ± 0.2	<8	-	33 ± 1	2.35	4.7 ± 0.4	NA	NA	NA	NA
		**Sand**	3.5 ± 0.6	-	75 ± 4	-	2.36	5.0 ± 0.1	-	<0.006	<0.2	<0.8
	**RCA2**	**Gravel**	1.5 ± 0.4	<8	-	30 ± 1	2.28	5.7 ± 0.4	<0.4	NA	NA	NA
		**Grit**	<0.5	<8	-	27 ± 1	2.31	5.4 ± 0.4	NA	NA	NA	NA
		**Sand**	3.1 ± 0.5	-	76 ± 4	-	2.23	7.3 ± 0.1	-	<0.006	<0.2	<0.8
	**RCA3**	**Gravel**	1.8 ± 0.4	<8	-	32 ± 1	2.21	6.4 ± 0.4	<0.4	NA	NA	NA
		**Grit**	1.6 ± 0.4	<8	-	28 ± 1	2.25	6.0 ± 0.4	NA	NA	NA	NA
		**Sand**	1.7 ± 0.4	-	90 ± 5	-	2.19	7.0 ± 0.1	-	NA	0.2 ± 0.1	<0.8
	**RSCCA1**	**Gravel**	2.1 ± 0.4	<8	-	36 ± 1	2.23	6.4 ± 0.4	<0.4	NA	NA	NA
		**Grit**	1.7 ± 0.4	<8	-	33 ± 1	2.25	6.3 ± 0.4	NA	NA	NA	NA
		**Sand**	1.9 ± 0.4	-	94 ± 1	-	2.29	6.5 ± 0.1	-	0.007	<0.2	<0.8
	**RSCCA2**	**Gravel**	2.6 ± 0.5	<8	-	32 ± 1	2.23	6.9 ± 0.4	<0.4	NA	NA	NA
		**Grit**	2.1 ± 0.4	<8	-	27 ± 1	2.28	6.2 ± 0.4	NA	NA	NA	NA
		**Sand**	1.6 ± 0.4	-	95 ± 5	-	2.28	6.5 ± 0.1	-	<0.006	0.2 ± 0.1	<0.8
	**RSCCA3**	**Gravel**	2.9 ± 0.5	<8	-	34 ± 1	2.18	7.7 ± 0.4	<0.4	NA	NA	NA
		**Grit**	2.4 ± 0.5	<8	-	29 ± 1	2.22	6.9 ± 0.4	NA	NA	NA	NA
		**Sand**	2.5 ± 0.5	-	98 ± 5	-	2.17	6.8 ± 0.1	-	<0.006	<0.2	<0.8

## Data Availability

Data are contained within the article.
